# Editorial: A brief Food & Nutrition Research status update

**DOI:** 10.3402/fnr.v60.33092

**Published:** 2016-08-24

**Authors:** Asim K. Duttaroy

**Affiliations:** Food & Nutrition Research

*Food & Nutrition Research* (FNR) was recently awarded a very high Impact Factor of 3.226, making it one of the top journals serving nutrition scientists and researchers. I hope *FNR*'s high Impact Factor will contribute to an even greater number of high-quality submissions on nutrition from around the world. The increase in the journal's Impact Factor is the result of the combined efforts of the authors who chose to publish their good work in this journal, as well as those of the editors, editorial board members, and reviewers, along with the team managing the journal. I congratulate all of these individuals and thank them for their invaluable contributions.

In addition to a new Impact Factor, the journal has undergone several changes related to editorial content and now publishes on a wide variety of nutrition- and food-related topics, with papers from every corner of the globe. Indeed, over the last two years, there has been a surge in the number of submissions from beyond Europe, making it a truly global journal. In this context, I am very pleased to announce that some eminent nutritionists have accepted our invitation to write commentaries on topical issues of the day.

No doubt we are all very pleased with the increased popularity of the journal among nutritionists worldwide and the recent changes that we have made to further strengthen *FNR* editorially. The increase in the Impact Factor along with the worldwide popularity of *FNR* has certainly leant us a renewed energy as we work to further develop *FNR*. Among other changes, is a rapid completion of the review process and publication of articles once accepted. As always, *FNR* papers can be downloaded from any corner of the world, making it stand out among nutrition journals.

In addition to the above, an expansion of the editorial team is underway. The rapid increase in the number of manuscripts as well as the diverse nature of submissions has brought forth the need to revitalize and renew the editorial board as well as to recruit new section editors with expertise in diverse nutrition fields. Announcements regarding these items are forthcoming.

This is my first Editorial that I write as an Editor-In-Chief of *Food & Nutrition Research*. I am truly honored and privileged to serve the International Nutrition Community as Editor-In-Chief of *FNR*.

*Asim K. Duttaroy* Editor-In-Chief *Food & Nutrition Research*

